# Nitric Oxide Derived from Cytoglobin-Deficient Hepatic Stellate Cells Causes Suppression of Cytochrome *c* Oxidase Activity in Hepatocytes

**DOI:** 10.1089/ars.2021.0279

**Published:** 2023-03-16

**Authors:** Yoshinori Okina, Misako Sato-Matsubara, Yasutoshi Kido, Hayato Urushima, Atsuko Daikoku, Chiho Kadono, Yu Nakagama, Yuko Nitahara, Truong Huu Hoang, Le Thi Thanh Thuy, Tsutomu Matsubara, Naoko Ohtani, Kazuo Ikeda, Katsutoshi Yoshizato, Norifumi Kawada

**Affiliations:** ^1^ Department of Medical Biochemistry, Graduate School of Medicine, Osaka Metropolitan University, Osaka, Japan.; ^2^ Department of Hepatology, Graduate School of Medicine, Osaka Metropolitan University, Osaka, Japan.; ^3^ Endowed Laboratory of Synthetic Biology, Graduate School of Medicine, Osaka Metropolitan University, Osaka, Japan.; ^4^ Department of Virology and Parasitology, Graduate School of Medicine, Osaka Metropolitan University, Osaka, Japan.; ^5^ Research Center for Infectious Disease Sciences, Graduate School of Medicine, Osaka Metropolitan University, Osaka, Japan.; ^6^ Department of Anatomy and Regenerative Biology, Graduate School of Medicine, Osaka Metropolitan University, Osaka, Japan.; ^7^ Department of Pathophysiology, Graduate School of Medicine, Osaka Metropolitan University, Osaka, Japan.; ^8^ BioIntegrence Co., Ltd., Osaka, Japan.

**Keywords:** liver, mitochondrial complex, cell–cell interaction, superoxide, globin

## Abstract

**Aims:**

Cell–cell interactions between hepatocytes (Hep) and other liver cells are key to maintaining liver homeostasis. Cytoglobin (CYGB), expressed exclusively by hepatic stellate cells (HSC), is essential in mitigating mitochondrial oxidative stress. CYGB absence causes Hep dysfunction and evokes hepatocarcinogenesis through an elusive mechanism. CYGB deficiency is speculated to hinder nitric oxide dioxygenase (NOD) activity, resulting in the elevated formation and release of nitric oxide (NO). Hence, we hypothesized that NO accumulation induced by the loss of NOD activity in CYGB-deficient HSC could adversely affect mitochondrial function in Hep, leading to disease progression.

**Results:**

NO, a membrane-permeable gas metabolite overproduced by CYGB-deficient HSC, diffuses into the neighboring Hep to reversibly inhibit cytochrome *c* oxidase (CcO), resulting in the suppression of respiratory function in an electron transport chain (ETC). The binding of NO to CcO is proved using purified CcO fractions from *Cygb* knockout (*Cygb*^−*/*−^) mouse liver mitochondria. Its inhibitory action toward CcO-specific activity is fully reversed by the external administration of oxyhemoglobin chasing away the bound NO. Thus, these findings indicate that the attenuation of respiratory function in ETC causes liver damage through the formation of excessive reactive oxygen species. Treating *Cygb*^−/−^ mice with an NO synthase inhibitor successfully relieved NO-induced inhibition of CcO activity *in vivo*.

**Innovation and Conclusion:**

Our findings provide a biochemical link between CYGB-absence in HSC and neighboring Hep dysfunction; mechanistically the absence of CYGB in HSC causes mitochondrial dysfunction of Hep *via* the inhibition of CcO activity by HSC-derived NO. *Antioxid. Redox Signal.* 38, 463–479.

## Introduction

Hepatic stellate cells (HSC), liver-specific pericytes that are located in Disse's space of the hepatic sinusoid and in close contact with hepatocytes (Hep), represent 5%–8% of the overall liver cellular composition (Weiskirchen and Tacke, 2014), and act as guardians of Hep to maintain albumin and cytochrome P450 (CYP450) expression (Abu-Absi et al, 2004; Rojkind et al, 1995). While constituting only a minor population (Wake, 2006), HSC are known to play key roles in hepatic pathogenesis, both directly as an effector cell and indirectly through cellular interactions mediated by secreted mediators (Thompson et al, 2015).

**Innovation**

**FIG. 1.**
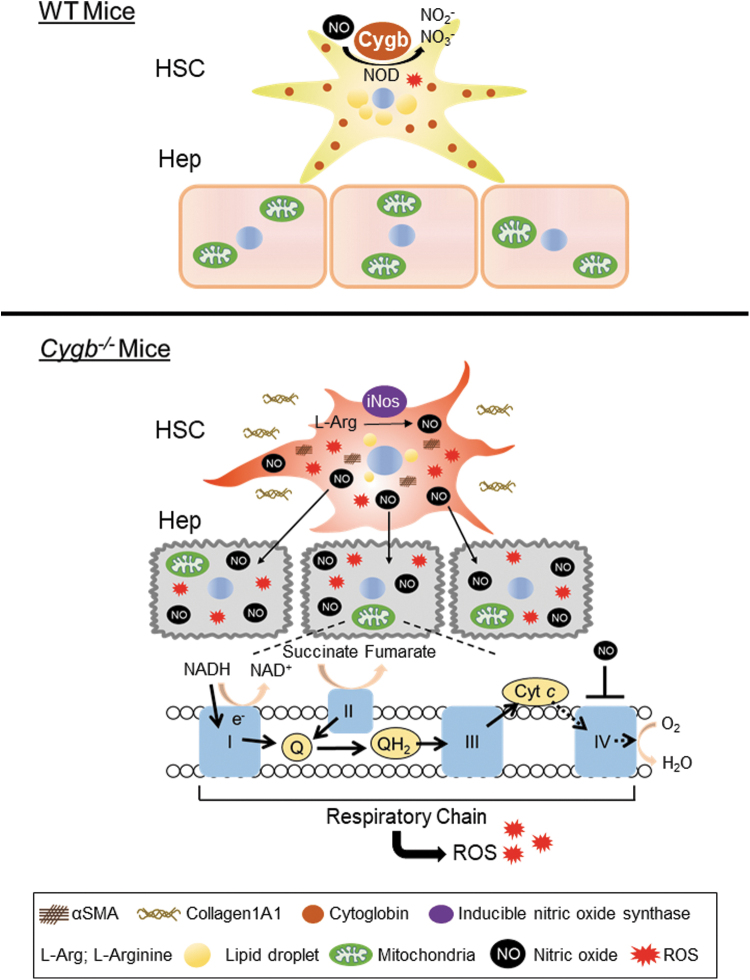
**Schematic illustration of the hypothesis proposed in this study.** HSC^*Cygb*−/−^ are more prone to activation than HSC^WT^. Intracellular NO accumulates in HSC^*Cygb*−/−^ due to the lack of NOD activity. NO released by HSC^*Cygb*−/−^ diffuses to neighboring Hep, affecting their mitochondrial function, and leading to attenuation of cytochrome *c* oxidase activity and induction of ROS production. CYGB, cytoglobin; *Cygb*^−/−^, *Cygb* knockout; Hep, hepatocytes; HSC, hepatic stellate cells; NO, nitric oxide; NOD, NO dioxygenase; ROS, reactive oxygen species; WT, wild type.

Hepatic stellate cells (HSC) are liver-specific pericytes lying in close interact with hepatocytes (Hep), which, while representing only a minority of the liver cell population, play key roles to maintaining liver function. Cytoglobin (CYGB), expressed exclusively by HSC, is known to possess nitric oxide (NO) dioxygenase activity. In the current study, we demonstrate the novel molecular mechanism by which CYGB-deficient HSC induce inhibition of cytochrome *c* oxidase activity *via* cell–cell interaction mediated by NO, resulting in dysfunction of Hep. Thus, our findings expand the scope of metabolite-mediated cell–cell interactions, and suggest a novel target for liver disease therapeutics (Fig. 1).

In the most well-known example of this cross talk between HSC and Hep, the aberrant secretion of extracellular matrix (ECM) by activated HSC has been proposed to contribute to the pathogenesis of cirrhosis (Barry et al, 2020). Indeed, activated HSC, characterized by the expression of α-smooth muscle actin (αSMA), promote liver fibrosis through the oversecretion of ECM, including type I collagen in the stroma and fibrogenic transforming growth factor-beta (TGF-β) (Dat et al, 2021; Okina et al, 2020).

Cytoglobin (CYGB), which is expressed exclusively in HSC in the liver, is a recently identified globin distinct from hemoglobin (Hb), myoglobin, neuroglobin, and androglobin (Burmester et al, 2002; Kawada et al, 2001). Our previous studies using mouse models of liver disease induced by a choline-deficient, L-amino acid-defined diet or by diethylnitrosamine administration elucidated that CYGB deficiency accelerated oxidative stress, liver inflammation, and fibrosis, and the development of hepatocellular carcinoma (HCC), accompanied by the activation of HSC (Thuy et al, 2015; Thuy et al, 2011). In contrast, the overexpression of CYGB in HSC, either in specific Cygb transgenic mice or through recombinant CYGB injection, hampered the activation of HSC, attenuating lipid peroxidation and oxidative DNA damage in Hep and consequently suppressing liver fibrosis (Dat et al, 2021; Thi Thanh Hai et al, 2018). Nonetheless, the molecular mechanism by which HSC activation in CYGB deficiency gives rise to Hep dysfunction remains undetermined.

CYGB possesses not only the capacity to bind oxygen (O_2_)_,_ carbon monoxide, and nitric oxide (NO) similar to other globins (Liu et al, 2017; Sawai et al, 2005) but also NO dioxygenase (NOD) activity, an essential function that converts NO to innocuous nitrate by the incorporation of molecular oxygen into NO in an O_2_-dependent manner (Zhou et al, 2017). Thus, CYGB deficiency is speculated to hinder NOD activity, resulting in elevated formation and release of NO. Hence, we hypothesized that NO accumulation induced by the loss of NOD activity in CYGB-deficient HSC could adversely affect mitochondrial function in Hep, leading to disease progression.

In this study, we quantify the amounts of NO and reactive oxygen species (ROS) in CYGB-deficient HSC, and we evaluate the mitochondrial respiratory chain activity *in vivo* and *in vitro*. This finding may contribute a missing clue to solving the question of how activated HSC trigger dysfunction of Hep and carcinogenesis *via* cell–cell interaction.

## Results

### CYGB absence induces excessive HSC NO production

**FIG. 2.**
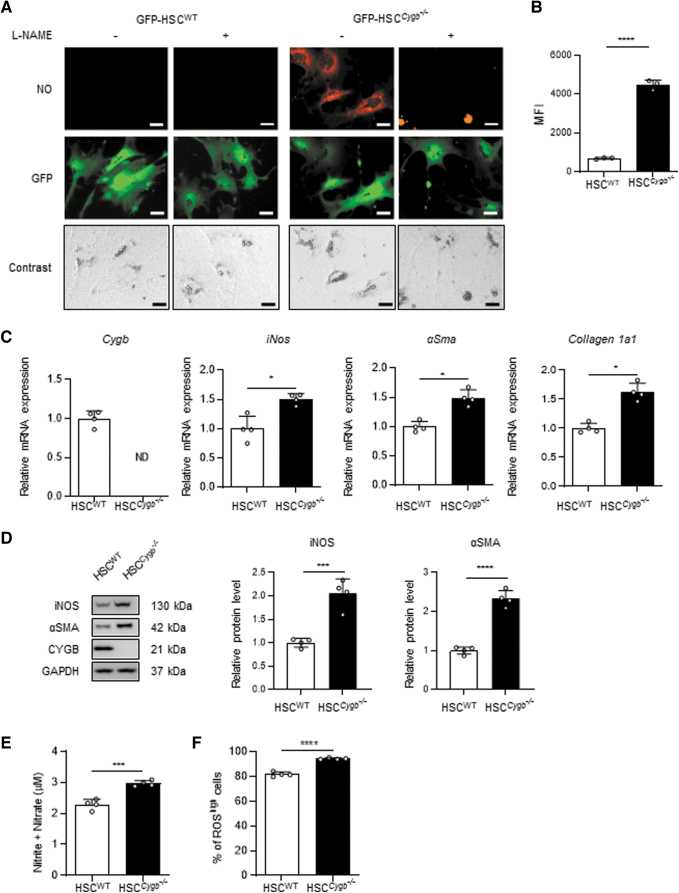
**Accumulation of intracellular NO in CYGB-absent HSC. (A)** Primary mouse HSC isolated from GFP-positive WT (HSC^WT^) or *Cygb*^−/−^ mice (HSC^*Cygb*−/−^) were plated on collagen-coated culture dishes, and incubated in the presence or absence of 3 m*M* L-NAME for 72 h. The intracellular NO level (*orange*) was evaluated using 5 μ*M* DAR-4M AM in GFP-positive (*green*) HSC^WT^ and HSC^*Cygb*−/−^. Scale bars, 20 μm. **(B)** Quantification of intracellular NO in HSC^WT^ and HSC^*Cygb*−/−^ by flow cytometry analysis. **(C)** Relative mRNA expression *of Cygb*, *iNos*, *αSma*, and *Collagen 1a1* in HSC^WT^ and HSC^*Cygb*−/−^ isolated from the livers of WT and *Cygb*^−/−^ mice. **(D)** Protein expression *of CYGB*, αSMA, and iNOS in HSC^WT^ and HSC^*Cygb*−/−^. GAPDH was used as a loading control. Bar graphs show relative fold change normalized to GAPDH compared with HSC^WT^. **(E)** The total concentration of nitrite/nitrate in media from HSC^WT^ and HSC^*Cygb*−/−^ cultures was quantified with timing identical to that in Figure 2b. **(F)** Quantification of intracellular ROS-dependent CM-H_2_DCFDA fluorescence in HSC^WT^ and HSC^*Cygb*−/−^ by flow cytometry analysis. Data are expressed as mean ± SD, *n* = 4. *p < 0.05, ***p < 0.001, ****p < 0.0001 by two-tailed, unpaired Student's *t*-test. αSMA, α-smooth muscle actin; CM, conditioned medium; DAR-4M AM, diaminorhodamine-4M acetoxymethyl ester; GAPDH, glyceraldehyde-3-phosphate dehydrogenase; iNOS, inducible NO synthase; L-NAME, N^ω^-nitro-L-arginine methyl ester hydrochloride; MFI, mean fluorescence intensity; mRNA, messenger RNA; ND, not detected; SD, standard deviation.

Considering the known NOD activity of CYGB (Zhou et al, 2017), we examined intracellular NO levels in primary mouse HSC using the NO-specific fluorescent probe diaminorhodamine-4M acetoxymethyl ester (DAR-4M AM). Hereafter, HSC isolated from wild-type (WT) and *Cygb* knockout (*Cygb*^−/−^) mice are designated HSC^WT^ and HSC^*Cygb*−/−^, respectively. Primary mouse HSC (1.5 × 10^5^ cells/mL) were plated on collagen-coated culture dishes and incubated for 72 h in the presence or absence of 3 m*M* nitric oxide synthase (NOS) inhibitor, N^ω^-nitro-L-arginine methyl ester hydrochloride (L-NAME). NO-specific signal was observed in HSC^*Cygb*−/−^, but not in HSC^WT^ (Fig. 2A). The level of NO was significantly decreased in L-NAME-treated HSC^*Cygb*−/−^ (Fig. 2A).

Flow cytometry analysis also showed that the intracellular NO level was significantly increased, by 6.4-fold, in HSC^*Cygb*−/−^ compared with HSC^WT^ (Fig. 2B). HSC devoid of *Cygb* messenger RNA (mRNA) expression showed a 1.7-fold increase in inducible NO synthase (*iNOS*) mRNA, relative to HSC^WT^, in parallel with markers of HSC activation such as *αSMA* and *Collagen 1a1* mRNA (Fig. 2C). Protein expression of iNOS and αSMA in HSC^*Cygb*−/−^ was also increased 2-fold and 2.3-fold, respectively, compared with HSC^WT^ (Fig. 2D). The concentration of NO in culture medium, quantified by its complete oxidation to nitrite and nitrate, was increased 1.3-fold in HSC^*Cygb*−/−^ compared with HSC^WT^ (Fig. 2E). These data indicated that HSC^*Cygb*−/−^ exhibited overproduction and secretion of NO through iNOS upregulation, converting the cells to the active state. Furthermore, we used flow cytometry analysis to assess intracellular ROS production in HSC^WT^ and HSC^*Cygb*−/−^.

In accordance with a previous result (Thuy et al, 2015), the population of ROS^high^ cells was expanded in HSC^*Cygb*−/−^ (94%) compared with HSC^WT^ (81%) (Fig. 2F). NO produced by iNOS serves as the major source of reactive nitrogen species, and contributes to pathological processes in various organs (Di Meo et al, 2016). Therefore, we assumed that large amounts of NO accumulated by HSC would be released extracellularly, and could have detrimental effects on the neighboring Hep.

### NO from HSC^*Cygb*−/−^ suppresses Hep cytochrome P450 1A2 activity

**FIG. 3.**
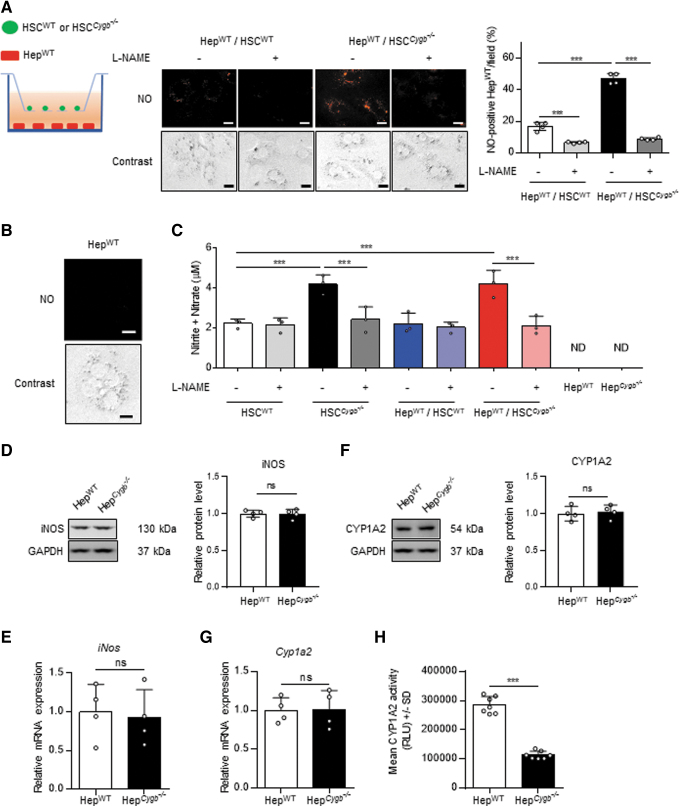
**NO released from HSC**^***Cygb***−**/**−^ **accumulating in cocultured Hep^WT^. (A)** The schematic image at *left* illustrates the coculture system that we introduced to mimic the *in vivo* microenvironment, in which HSC^WT^ or HSC^*Cygb*−/−^ were cultured in the insert along with Hep^WT^ in the lower well. HSC^WT^ and HSC^*Cygb*−/−^ were plated on a cell culture insert with pore size of 1.0 μm, and maintained with the presence or absence of 3 m*M* L-NAME. After 72 h, each cell line was cocultured with Hep^WT^. Intracellular NO (*orange*) in Hep^WT^ was evaluated using 5 μ*M* DAR-4M AM. Scale bars, 20 μm. The graph at *right* shows the percentage of NO-positive Hep^WT^ per field. **(B)** Intracellular NO in Hep^WT^ monoculture. Scale bars, 20 μm. **(C)** The total concentration of nitrite/nitrate was quantified in cultured media from HSC^WT^ and HSC^*Cygb*−/−^ monocultures, from Hep^WT^ cocultured with HSC^WT^ or HSC^*Cygb*−/−^, and from Hep^WT^ and Hep^*Cygb*−/−^ monocultures. **(D, E)** Protein and mRNA expression *of iNOS* in Hep^WT^ and Hep^*Cygb*−/−^. GAPDH was used as a loading control. Bar graphs show relative fold change normalized to GAPDH compared with Hep^WT^. **(F, G)** Protein and mRNA expression *of CYP1A2* in Hep^WT^ and Hep^*Cygb*−/−^. GAPDH was used as a loading control. Bar graphs show relative fold change normalized to GAPDH compared with Hep^WT^. **(H)** The activity of CYP1A2 in Hep^WT^ and Hep^*Cygb*−/−^ was quantified with Luciferin-ME as substrate. Data are expressed as mean ± SD, *n* = 4. ***p < 0.001 by one-way ANOVA **(A, C)** or two-tailed unpaired Student's *t*-test **(D–H)**. ANOVA, analysis of variance; CYP1A2, cytochrome P450 1A2; ns, not significant; RLU, relative light units.

We then investigated whether NO derived from HSC diffuses to the neighboring Hep. Hereafter, Hep isolated from WT and *Cygb*^−/−^ mice are designated Hep^WT^ and Hep^*Cygb*−/−^, respectively. To mimic the *in vivo* microenvironment, we introduced a coculture system in which HSC^WT^ or HSC^*Cygb*−/−^ were cultured in inserts along with Hep^WT^ (Fig. 3A). Following a 72-h preculture with or without 3 m*M* L-NAME, HSC-containing inserts (1.0 × 10^5^ cells/mL) were washed with phosphate-buffered saline (PBS) and transferred to wells with Hep^WT^ (0.5 × 10^5^ cells/mL) to establish the coculture system (Fig. 3A). After 48 h, the proportion of NO-positive Hep^WT^ cells was significantly increased 2.8-fold in Hep^WT^ cocultured with HSC^*Cygb*−/−^, compared with Hep^WT^ cocultured with HSC^WT^ (Fig. 3A). Moreover, pretreatment of the HSC with L-NAME attenuated the NO accumulation in the cocultured Hep^WT^. NO-positive cells were not detected in Hep^WT^ monoculture (Fig. 3B). 

Next, we confirmed the NO levels (total nitrite and nitrate) in media collected from the cultures and cocultures. The NO level was increased 1.9-fold in HSC^*Cygb*−/−^ culture media, and also 1.9-fold in media from cocultures of Hep^WT^ with HSC^*Cygb*−/−^, compared with HSC^WT^ monoculture medium (Fig. 3C). NO was detected in cultured media of Hep^WT^ or Hep^*Cygb*−/−^ monocultures (Fig. 3C). Furthermore, both protein and mRNA levels of iNOS were unchanged between Hep^WT^ and Hep^*Cygb*−/−^ (Fig. 3D, E). These results demonstrate that NO released from HSC^*Cygb*−/−^ was transferred to neighboring Hep^WT^ in the microenvironment-mimicking system.

Next, to confirm that transferred NO retained its biological activity within Hep, we measured the activity of cytochrome P450 1A2 (CYP1A2) enzyme, which is known to be directly targeted by NO (Nakano et al, 1996; Stadler et al, 1994). First, neither protein nor mRNA levels of CYP1A2 differed between Hep^WT^ and Hep^*Cygb*−/−^ (Fig. 3F, G). However, CYP1A2 activity was significantly attenuated, by 2.5-fold, in Hep^*Cygb*−/−^ compared with Hep^WT^ (Fig. 3H). Taken together, these results show that the excessive NO accumulated in HSC^*Cygb*−/−^ was released extracellularly and transferred to Hep, resulting in the suppression of CYP1A2 activity.

### Cytochrome *c* oxidase activity is specifically inhibited in *Cygb*^−/−^ mice

**FIG. 4.**
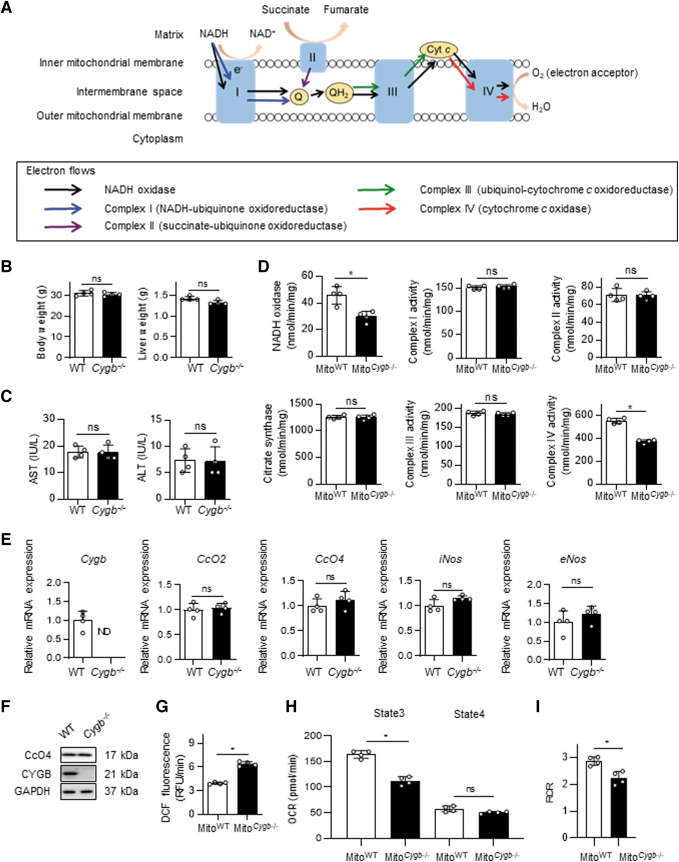
**Enzymatic analysis of respiratory chain function in mitochondria fractionated from the livers of WT and *Cygb***^−**/**−^ **mice. (A)** Schematic image of the respiratory chain in mitochondria. Complexes I–IV are marked as I, II, III, and IV, respectively. Each *arrow* indicates the electron flow. **(B)** Body weights (g) and liver weights (g) of WT and *Cygb*^−/−^ mice. **(C)** Serum levels of AST (IU/L) and ALT (IU/L) in WT and *Cygb*^−/−^ mice. **(D)** Each *panel* exhibits the specific enzyme activity of NADH oxidase, citrate synthase, complex I, complex II, complex III, and complex IV in the mitochondria fractionated from WT (Mito^WT^) and *Cygb*^−/−^ mice (Mito^*Cygb*−/−^). **(E)** Relative mRNA expression of *Cygb*, *CcO2*, *CcO4*, *iNos*, and *eNos* in the livers of WT and *Cygb*^−/−^ mice. **(F)** CYGB and CcO4 protein expression in the livers of WT and *Cygb*^−/−^ mice. CYGB protein was ND in *Cygb*^−/−^ mice. No difference was observed in CcO4 protein expression between WT and *Cygb*^−/−^ mice. GAPDH was used as a loading control. **(G)** Mitochondrial ROS detected in Mito^WT^ and Mito^*Cygb*−/−^. Kinetic measurements were performed for 10 min and DCF fluorescence was recorded at 30-s intervals using a microplate reader. **(H, I)** Respiration in isolated mitochondria was analyzed using a Seahorse XF HS Mini Analyzer. State 3, state 4, and RCR were measured by using succinate, ADP, oligomycin, and FCCP, respectively. Rotenone and antimycin A were included to record nonmitochondrial respiration. Data are expressed as mean ± SD, *n* = 4. *p < 0.05 by two-tailed, unpaired Student's *t*-test. ALT, alanine aminotransferase; AST, aspartate aminotransferase; CcO, cytochrome *c* oxidase; Cyt *c*, cytochrome *c*; e^−^, electron; eNOS, endothelial nitric oxide synthase; FCCP, carbonyl cyanide 4-(trifluoromethoxy) phenylhydrazone; Mito, mitochondria; NADH, nicotinamide adenine dinucleotide; Q, ubiquinone; QH_2_, ubiquinol; RCR, respiratory control ratio.

The fact that NO released by HSC^*Cygb*−/−^ was transferred to neighboring Hep^WT^ (Fig. 3A) raises the question of how NO impacts the cell–cell interaction between HSC and Hep. Because NO is a well-known competitive inhibitor of mitochondrial CcO with respect to O_2_ (Brookes et al, 2003; Brown, 2001), we evaluated electron transport chain (ETC) function of mitochondria fractionated from the liver of WT and *Cygb*^−/−^ mice (Mito^WT^ and Mito^*Cygb*−/−^, respectively) (Fig. 4A). Body weight and liver weight were similar between the two groups of mice (Fig. 4B), as were serum levels of aspartate aminotransferase (AST) and alanine aminotransferase (ALT) (Fig. 4C). However, we clearly demonstrated that nicotinamide adenine dinucleotide (NADH) oxidase activity and CcO activity of complex IV in Mito^*Cygb*−/−^ were significantly attenuated, by 35% and 32%, respectively, compared with Mito^WT^ (Fig. 4D).

In contrast, the specific activities of complex I (NADH-ubiquinone oxidoreductase), II (succinate-ubiquinone oxidoreductase), and III (ubiquinol-cytochrome *c* oxidoreductase) were comparable between Mito^WT^ and Mito^*Cygb*−/−^ (Fig. 4D). Furthermore, the specific activity of citrate synthase, a functional marker of the tricarboxylic acid cycle, was similar between Mito^WT^ and Mito^*Cygb*−/−^ (Fig. 4D). We confirmed that hepatic *Cygb* expression was not detected in *Cygb*^−/−^ mice, and that no differences existed in the hepatic expression of *CcO2*, *CcO4*, *iNos*, or *eNos* mRNA between WT and *Cygb*^−/−^ mice (Fig. 4E). In addition, the hepatic protein expression level of CcO4 was also unaltered between WT and *Cygb*^−/−^ mice (Fig. 4F). Mito^*Cygb*−/−^ displayed induction of mitochondrial ROS production compared with Mito^WT^ (Fig. 4G).

Mitochondrial respiratory chain activity was analyzed for state 3 respiration, state 4 respiration, and the respiratory control ratio (RCR), with the addition of various combinations of substrates and inhibitors. State 3 respiration was significantly reduced in Mito^*Cygb*−/−^, compared with Mito^WT^, while state 4 respiration was unchanged between Mito^WT^ and Mito^*Cygb*−/−^. Moreover, the RCR was significantly reduced in Mito^*Cygb*−/−^, compared with Mito^WT^. Taking these results together, we consider that the excess NO released by CYGB-ablated HSC inhibits CcO activity of Hep.

### NO competitively inhibits purified CcO from Mito^*Cygb*−/−^

**FIG. 5.**
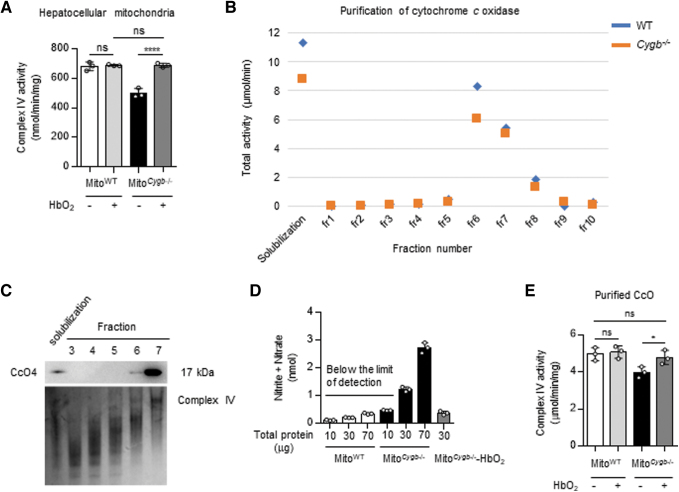
**NO detected in fractionated mitochondria and purified CcO. (A)** CcO activity in Mito^*Cygb*−/−^ was attenuated at baseline. Upon the addition of HbO_2_, the specific activity of CcO recovered to a level comparable with that of Mito^WT^. **(B)** DDM-solubilized mitochondria were further fractionated by 0.5–1.5 *M* sucrose gradient ultracentrifugation. Fractions 1–10 (1 mL each) were collected, starting from the top of the gradient. The distribution of total CcO activity in each fraction is shown. **(C)** The purity of CcO in each fraction was analyzed by Western blot and clear-native PAGE. Representative images of CcO4 expression detected by Western blotting (*upper*) and complex IV abundance detected by clear-native PAGE (*lower*) in the respective fractions are shown. **(D)** The total concentration of nitrite/nitrate was quantified in CcO-purified fractions from Mito^WT^ and Mito^*Cygb*−/−^. The amount of total nitrite/nitrate was below the limit of detection in CcO purified from Mito^WT^. **(E)** Attenuation of CcO activity in Mito^*Cygb*−/−^ was confirmed using the CcO-purified mitochondrial fraction. By the addition of HbO_2_, the specific activity of CcO purified from Mito^*Cygb*−/−^ was restored to a level comparable with the fraction purified from Mito^WT^. Data are expressed as mean ± SD, *n* = 3. *p < 0.05, ****p < 0.0001 by one-way ANOVA. DDM, n-dodecyl-β-D-maltopyranoside; HbO_2_, oxyhemoglobin; PAGE, polyacrylamide gel electrophoresis.

Under the assumption that NO released by CYGB-absent HSC would directly inhibit CcO activity in Hep, NO bound to CcO should be displaced by the addition of oxyhemoglobin (HbO_2_), as expressed in the following equation: cytochrome *c* oxidase-NO + HbO_2_ ⇄ cytochrome *c* oxidase-O_2_ + NO + Hb. When 3 μg of HbO_2_ was mixed with 10 μg of mitochondrial fraction for 5 min on ice to chase away NO from its binding site in complex IV, the activity of CcO of Mito^*Cygb*−/−^ was fully restored to that of Mito^WT^ (Fig. 5A). To prove that the observed phenomenon was underlaid by the above chemical relationship, we next attempted to detect bound NO in CcO purified from Mito^WT^ and Mito^*Cygb*−/−^ by solubilization with n-dodecyl-β-D-maltopyranoside (DDM) and fractionation by sucrose gradient ultracentrifugation. Representative data are shown in Figure 5B.

As summarized in Supplementary Table S1, the highest specific CcO activities were observed in the purified fraction 7 of both Mito^WT^ (5.32 μmol/min/mg) and Mito^*Cygb*−/−^ (4.28 μmol/min/mg). The purity of the samples was checked by Western blot and clear-native polyacrylamide gel electrophoresis (PAGE) (Fig. 5C). We quantified total NO in fraction 7 for both groups (Fig. 5B and Supplementary Table S1). We successfully detected 1.31 and 2.73 nmol of NO in 30 and 70 μg, respectively, of the purified CcO fraction of Mito^*Cygb*−/−^ (Fig. 5D), while NO was not detected in the CcO fraction of Mito^WT^. The addition of 5 μg HbO_2_ to the purified CcO fraction of Mito^*Cygb*−/−^ again released NO from its binding site, so that total NO was no longer detected (Fig. 5D). Furthermore, specific CcO activity in the purified CcO of Mito^*Cygb*−/−^ was restored after HbO_2_ addition to the same level as that of Mito^WT^ (Fig. 5E).

These data strongly suggested that the NO released from HSC^*Cygb*−/−^ was reversely bound to CcO in Hep^*Cygb*−/−,^ and inhibited mitochondrial respiration (Brookes et al, 2003).

### L-NAME treatment restores CcO activity in *Cygb*^−/−^ mice

**FIG. 6.**
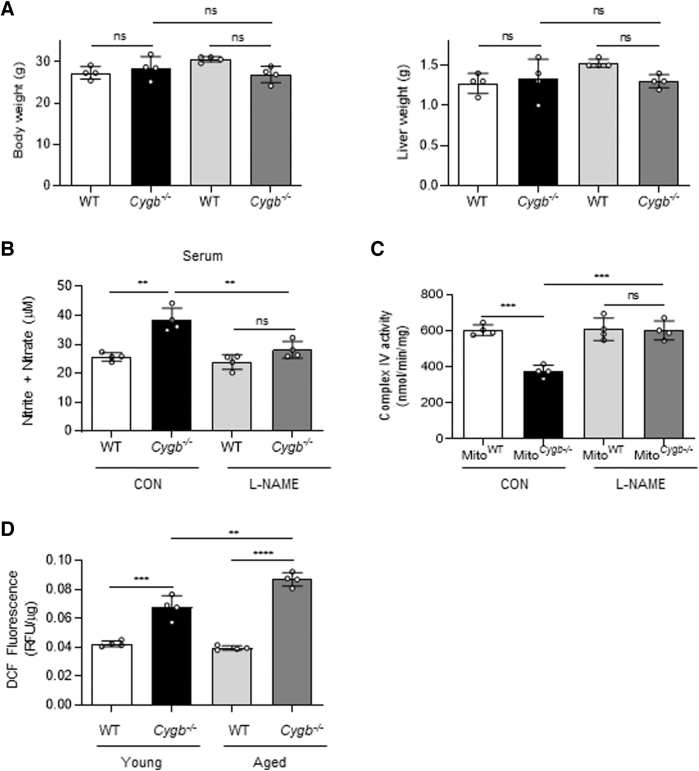
**L-NAME administration rescues *in vivo* hepatic CcO activity from attenuation by NO. (A)** L-NAME was administered intraperitoneally at 300 mg kg^−1^ per day for 4 days. Control mice (CON) were injected with PBS. *Left* and *right panels* show the body weights (g) and liver weights (g) of WT and *Cygb*^−/−^ mice, respectively. **(B)** The total concentration of nitrite/nitrate was quantified in serum of WT and *Cygb*^−/−^ mice. The amount of total nitrite and nitrate was attenuated in *Cygb*^−/−^ mice by L-NAME administration. **(C)** While having no effect on hepatic CcO activity of Mito^WT^, L-NAME administration reversed the NO-induced attenuation of hepatic CcO activity in Mito^*Cygb*−/−^. **(D)** ROS in liver tissue of young and aged WT and *Cygb*^−/−^ mice, detected with DCFDA. Young mice were 8–12 weeks of age, and aged mice were 42–45 weeks of age. DCF fluorescence was normalized per microgram of protein concentration. Data are expressed as mean ± SD, *n* = 4. **p < 0.01, ***p < 0.001, ****p < 0.0001 by two-way ANOVA. PBS, phosphate-buffered saline; RFU, relative fluorescence units.

Mice were administrated the NOS inhibitor L-NAME, to clarify the reversal of NO-induced inhibition of CcO activity in Hep^*Cygb*−/−^. After 4 days of intraperitoneal injection of L-NAME, or PBS in control mice (CON), no significant differences in body weight or liver weight were observed between the two groups (Fig. 6A). The NO level (total nitrite and nitrate) in serum of *Cygb*^−/−^ mice was significantly decreased, 1.4-fold, following L-NAME administration (Fig. 6B). Consistent with previous results (Fig. 4D), hepatic CcO activity of Mito^*Cygb*−/−^ was significantly attenuated compared with Mito^WT^ in the control group. However, hepatic CcO activity of Mito^*Cygb*−/−^ was reversed by L-NAME-administration to *Cygb*^−/−^ mice, leading to complete recovery of activity to a level comparable with Mito^WT^ (Fig. 6C). Finally, to further support our data, we examined intracellular ROS accumulation in liver tissue of WT and *Cygb*^−/−^ mice.

Hereafter, mice 8–12 weeks of age and 42–45 weeks of age are referred to as young and aged mice, respectively. ROS levels significantly increased 1.6-fold in young *Cygb*^−/−^ mice compared with young WT mice (Fig. 6D). Furthermore, aged *Cygb*^−/−^ mice displayed significantly greater ROS accumulation, 2.3-fold compared with aged WT mice, and 1.4-fold compared with young *Cygb*^−/−^ mice, (Fig. 6D).

## Discussion

The attenuation of CcO activity in Hep triggered by excessive NO released from HSC provides a molecular rationale for how activated, CYGB-deficient HSC facilitate the pathological dysfunction of Hep through cell–cell interaction, although caution must be taken in the interpretation of these snapshot data, because they were derived from biochemical experiments under nonphysiological conditions. A brief exposure to electrolysis-generated oxygen free radicals decreased agonist-induced NO release in isolated rat hearts (Paolocci et al, 2001). Nevertheless, NO was substantially detected in a dose-dependent manner in the purified CcO fraction isolated from Mito^*Cygb*−/−^, but not from Mito^WT^ (Fig. 5D), which indicated the presence of excess amounts of NO in the microenvironment composed of HSC and Hep in *Cygb*^−/−^ mice.

By comparing a calculated *k*_cat_ of 14.3 s^−1^ in the purified CcO fraction from Mito^*Cygb*−/−^ (Supplementary Table S1) with the value calculated for cytochrome *c* oxidase (CcO; 200–600 s^−1^) (Vygodina et al, 2013), the apparent purity of this fraction can be estimated as 2.9%. Taking these values into account, the quantified amounts of NO would be assumed to stoichiometrically bind to 30% of total complex IV (Fig. 5B). To solidify our model, we must further investigate whether inhibition of the CcO enzyme by NO causes ROS accumulation in the long term using *Cygb*^−/−^ mice.

The mitochondrial respiratory chain is the major source of electron leak, and thus ROS production, within mammalian cells (Li et al, 2013). Electron leak and ROS production from the respiratory chain occur when electrons exit the chain before the terminal CcO step (Jastroch et al, 2010). Through extensive comparison of the enzymatic activities of all respiratory chain components, we here have demonstrated that complex IV activity alone was disproportionately attenuated in Mito^*Cygb*−/−^ (Fig. 4D). NO-inhibited mitochondria produce superoxide (Thomas et al, 2001). We have also detected ROS in isolated mitochondria in liver tissues (Fig. 4G). Such stagnation of electron flow in the milieu of the respiratory complexes serves as the biochemical basis for electron leakage, which in turn may possibly represent the source of mitochondrial ROS production in *Cygb*^−/−^ mice (Fig. 6D).

The bioactive gas NO is produced from L-arginine during its conversion to citrulline, an NOS-catalyzed reaction (Iwakiri and Kim, 2015). Moreover, NO is able to undergo radical–radical reaction with O_2_^-^ in mitochondria to form peroxynitrite, which induces DNA damage and disruption of mitochondrial integrity, and may also play a crucial role in carcinogenesis (Li et al, 2013). In the liver, not only HSC but also Hep, Kupffer cells, and endothelial cells have the capacity to generate NO and become NO donors (Laskin et al, 2001). With the efficient coordination of NO scavenger cells, NO signaling in the proximity of NOS is kept compartmentalized to maintain homeostasis in the tissue microenvironment. Ectopic NO due to spillover and excessive diffusion is considered to provoke liver damage, along with augmented inflammation and fibrosis induction in patients infected with hepatitis C virus (Tache et al, 2014).

The inhibition of CYP1A2 enzyme activity mediated by NO caused liver H_2_O_2_ production, resulting in Hep dysfunction (Shertzer et al, 2004). By inducing DNA damage or hindering DNA repair, iNOS is associated with the pathophysiology of inflammatory disorders in human cholangiocytes (Jaiswal et al, 2000). Both alcoholic steatohepatitis and nonalcoholic steatohepatitis are associated with increased sensitivity of the respiratory chain to inhibition by NO, increased hypoxia, and protein nitration (Mantena et al, 2009; Shiva et al, 2005; Venkatraman et al, 2004). It is proposed that these NO-dependent changes result in excessive inhibition or altered regulation of the respiratory chain. These lead to bioenergetic dysfunction, reductive stress, and ROS production, all of which are key features of mitochondrial dysfunction in diabetes/obesity and alcohol-mediated liver disease.

Hormesis involving NO is a dose–response phenomenon characterized by a low-dose stimulation and a high-dose inhibition (Calabrese et al, 2010a; Calabrese et al, 2010b; Calabrese et al, 2007). NO is involved in several cellular functions, including neurotransmission, the regulation of blood vessel tone, and the immune response. Excessive NO can interact with mitochondrial superoxide to generate the highly reactive free radical peroxynitrite (Calabrese et al, 2010a; Calabrese et al, 2010b; Calabrese et al, 2007).

The three pathological aspects of CcO dysfunction can be summarized as follows: (1) a biomarker for cancer initiation and progression; (2) ROS production; and (3) mitochondrial diseases. In category 1, mitochondrial DNA (mtDNA) sequence was mutated in 70% of human colorectal cancer cell lines analyzed, as well as in human colon cancer, and the mtDNA mutations may have arisen from ROS damage (Polyak et al, 1998). A previous study by Petros et al (2005) showed that 11%–12% of all prostate cancer patients had cytochrome oxidase subunit I mutations that altered conserved amino acids. In category 2, CcO may also contribute to lowering ROS levels due to its antioxidant properties (Korshunov et al, 1999; Pereverzev et al, 2003). Thus, a defect in CcO can cause elevated ROS levels, followed by increased mtDNA damage (Chatterjee et al, 2006).

In category 3, mitochondrial diseases are a clinically and genetically heterogeneous group of disorders that result from dysfunction of the ETC. The liver, being an actively biosynthetic and detoxifying organ, is one of the richest organs in terms of mitochondrial content (Degli Esposti et al, 2012). Deficiency of the CcO enzyme is the major cause of ETC dysfunction, leading to mitochondrial hepatopathies. Congenital deficiency of subunits that comprise the CcO enzymatic complex causes neonatal liver failure, steatohepatitis, or cholestasis of early childhood onset (Lee and Sokol, 2007). On the contrary, acquired CcO deficiency, which has previously been ascribed to the accumulation of mtDNA defects over time and the subsequent ETC disruption, may be central in the pathogenesis of a nonalcoholic fatty liver disease (Paradies et al, 2014). The inhibition of CcO, resulting in the suppression of mitochondrial respiratory chain dysfunction, causes liver damage through excessive ROS formation.

This aspect should also be considered in the relationship with hormesis involving low NO levels (Calabrese et al, 2010a; Calabrese et al, 2010b; Calabrese et al, 2007).

CYGB is also thought to be involved in the development of cancer. In most cancers, represented by esophageal and nonsmall-cell lung cancer, CYGB expression is downregulated through promoter hypermethylation (McRonald et al, 2012; McRonald et al, 2006; Shivapurkar et al, 2008). The downregulation of CYGB expression in cancer cells suggests that it plays a possible role as a tumor suppressor gene. Furthermore, most cancer cells have a reduced expression of CYGB, with a dramatic decrease (70%) of CYGB expression reported in tylosis with esophageal cancer (McRonald et al, 2006). The promotion of macroscopic tumor burden in an azoxymethane and dextran sulfate sodium-induced colorectal cancer were showed using *Cygb*^−*/*−^ mouse model (Yassin et al, 2018). We have also previously reported that *Cygb*^−*/*−^ mice exhibited spontaneous age-dependent malformations, and tumors in multiple organs (Thuy et al, 2016).

Because our present study showed mitochondrial dysfunction of *Cygb*^−*/*−^ mice at 12 weeks, we will further investigate the correlation of liver disease development and mitochondrial dysfunction in aged mice.

Regarding the cell–cell interaction between HSC and Hep in physiological conditions, HSC surround liver sinusoidal endothelial cells (LSECs) in normal livers, and their contact with LSECs is smooth and does not constitute direct cell adhesion. Conversely, HSC extend protrusions called spines, which establish adherens junctions with Hep-mediated E-cadherin and maintain HSC quiescence. In a normal liver, E-cadherin binding suppresses TAZ expression, which contributes to maintaining HSC quiescence. In contrast, the loss of this binding in an injured liver leads to an increase in TAZ expression and HSC activation. Thus, E-cadherin at the spines of quiescent HSC is a key component of the adherens junctions between Hep and HSC (Urushima et al, 2021).

Several studies investigated the effect of HSC on functions of HCC or cholangiocarcinoma cells by exposing the cancer cells to conditioned medium (CM) collected from activated HSC. This CM contains cytokines, including interleukin-10, TGF-β, Hep growth factor, and vascular endothelial growth factor, which induce cell proliferation, migration, and invasion of the cancer cells (Amann et al, 2009; Badiola et al, 2012; Gentilini et al, 2012; Rombouts et al, 2013). It is also well known that activated HSC infiltrate the HCC stroma and peritumoral tissue, and are localized around tumor sinusoids, fibrous septa, and the tumor capsule; therefore, activated HSC are among the key players in the formation of the tumor cell microenvironment (Dubuisson et al, 2001; Faouzi et al, 1999; Le Bail et al, 1999). This indicates that, besides playing a key role in fibrosis, HSC are of equal importance during HCC development and progression (Carloni et al, 2014).

CYGB expressed in HSC is a key player in the NO-scavenging reaction, and regulates the compartmentalization of NO signaling in the liver tissue microenvironment. Spillover of NO from HSC gives rise to a vicious cycle of the mitochondrial respiratory chain dysfunction-ROS production axis, through its biochemical inhibition of CcO in adjoining Hep. The excess ROS in the liver cause lipotoxicity and lead to mitochondrial dysfunction, inducing damage in Hep and inflammation (Paradies et al, 2014). Thus, gas-mediated modulation of the hepatic ETC opens a novel research area in hepatology, of deciphering the molecular players and druggable targets related to HSC-HCC cross talk.

In conclusion, the present study showed the NO fraction, overflowing from CYGB-absent HSC, and inhibited the CcO enzyme of Hep, leading to subsequent proton leak and ROS accumulation. Our data demonstrate that the NOD activity of CYGB is indispensable for keeping Hep healthy. Complementary NOD activity by CYGB expression may potentially lead to a novel therapeutic effect in diseases characterized by both oxidative and nitrosative stresses.

## Materials and Methods

Electronic laboratory notebook was not used. Uncropped images are shown in Supplementary Figure S1.

### Animal studies

*Cygb*^−/−^ mice (C57BL/6 background) were generated in our laboratory as described previously (Thuy et al, 2011). *Cygb*^−/−^ mice and their WT littermates (8–12 weeks of age and 42–45 weeks of age) were used in our experiments. GFP-positive *Cygb*^−/−^ mice were generated in our laboratory by mating *Cygb*^−/−^ mice with C57BL/6-Tg (*CAG*/Acr-*EGFP*) mice. The surgical procedures were performed under anesthesia *via* an intraperitoneal injection of 30 mg kg^−1^ body weight Somnopentyl (Kyoritsu Seiyaku Corp., Tokyo, Japan). For L-NAME (Sigma-Aldrich, St. Louis, MO) experiments, L-NAME was injected intraperitoneally at 300 mg kg^−1^ per day in PBS for 4 days. Control mice were injected with PBS. All protocols and experimental procedures were approved by the Institutional Animal Care and Use Committee of Osaka City University, and were performed following the guidelines of the National Institutes of Health (Bethesda, MD) for the use of animals in research.

### Isolation of primary mouse HSC and Hep

Primary mouse HSC were isolated in our laboratory from C57BL/6J mice (Japan SLC, Inc., Shizuoka, Japan) and *Cygb*^−*/*−^ mice using a pronase-collagenase digestion method as previously described (Okina et al, 2020). Briefly, normal livers were perfused for 3 min with an SC-1 solution consisting of 8000 mg/L NaCl, 400 mg/L KCl, 88.17 mg/L NaH_2_PO_4_·2H_2_O, 120.45 mg/L Na_2_HPO_4_, 2380 mg/L HEPES, 350 mg/L NaHCO_3_, 190 mg/L EGTA, and 900 mg/L glucose (pH 7.3). The livers were then digested at 37°C for 10 min with 0.1% pronase E (Merck Millipore, Billerica, MA), then for an additional 10 min with 0.05% collagenase (Wako Pure Chemical Industries Ltd., Osaka, Japan) dissolved in SC-2 solution consisting of 8000 mg/L NaCl, 400 mg/L KCl, 88.17 mg/L NaH_2_PO_4_·2H_2_O, 120.45 mg/L Na_2_HPO_4_, 2380 mg/L HEPES, 350 mg/L NaHCO_3_, and 560 mg/L CaCl_2_·2H_2_O (pH 7.3).

The digested livers were excised, cut into small pieces, and incubated at 37°C in SC-2 solution containing 0.04% pronase E, 0.04% collagenase, and 20 μg/mL DNase I (Roche Diagnostics, Mannheim, Germany). The resulting suspension was filtered through a 70 μm nylon cell strainer (Corning) and centrifuged on an 8.2% Nycodenz^®^ cushion (Axis-Shield PoC AS, Oslo, Norway), which produced an HSC-enriched fraction in the upper whitish layer. The cells were washed, suspended in Dulbecco's modified Eagle's medium (DMEM; Gibco, Waltham, MA) supplemented with 10% fetal bovine serum (FBS; Hyclone, Logan, UT) and antibiotics (100 U/mL penicillin and 100 mg/mL streptomycin), then plated on 24-well plastic culture dishes (Greiner Bio-One International GmbH, Kremsmünster, Austria), and incubated at 37°C in a 5% CO_2_ air environment. HSC purity was ∼95%, as assessed by the typical star-like shape with a lipid droplet configuration.

The cells were incubated overnight, then the culture medium was removed, and the cell layer was subsequently washed two times with PBS to remove dead cells and cellular debris. The adherent cells were used for experiments.

Primary mouse Hep were isolated from C57BL/6J mice and *Cygb*^−*/*−^ mice by *in situ* perfusion, using 30 mL of SC-1 solution and 30 mL of 0.05% collagenase solution. Then, cells were pelleted by centrifugation at 50 *g* for 4 min, three times. Primary mouse HSC and Hep were washed, suspended in DMEM supplemented with 10% FBS and antibiotics (100 U/mL penicillin and 100 mg/mL streptomycin), plated in 24-well plastic culture dishes (Greiner Bio-One International GmbH), and incubated at 37°C in a 5% CO_2_ environment.

### Coculture experiments

Primary mouse HSC isolated from WT and *Cygb*^−/−^ mice were plated on cell culture inserts with 1.0 μm pore size (Falcon; Becton Dickinson Labware). Following a 72-h preculture with or without 3 m*M* L-NAME, HSC-containing inserts (1.0 × 10^5^ cells/mL) were washed with PBS and transferred to wells containing Hep^WT^ (0.5 × 10^5^ cells/mL) and plated on the collagen-coated culture dishes (Iwaki; Asahi Glass. Co., Ltd., Tokyo, Japan).

### Western blot analyses

Proteins isolated from tissues or cells were lysed in radioimmunoprecipitation buffer containing protease inhibitors (Roche Diagnostics) and phosphatase inhibitors (Thermo Fisher Scientific, Waltham, MA). Proteins isolated from tissues (30 μg) or cells (4–6 μg) were separated by 5%–20% sodium dodecyl sulfate (SDS)-PAGE (DRC, Tokyo, Japan) using Precision Plus Protein^™^ Dual Color standards (Bio-Rad, Hercules, CA), and transferred to 0.45 μm polyvinylidene difluoride membranes (Bio-Rad). After blocking with 5% skim milk, the membranes were incubated with primary antibodies overnight at 4°C (Supplementary Table S2). The membranes were then incubated with horseradish peroxidase-conjugated goat anti-mouse or anti-rabbit secondary antibodies (1:4000; Dako; Agilent Technologies, Santa Clara, CA). Luminescence was quantified on an LAS-4000 luminescent image analyzer (Fujifilm Corp., Tokyo, Japan) coupled to image analysis software (Multi Gauge; Fujifilm Corp.). The staining intensity of glyceraldehyde-3-phosphate dehydrogenase (GAPDH) was used as a loading control.

### Quantitative RT-PCR

Total RNA was extracted from cells or liver tissues using the TRIzol reagent (Thermo Fisher Scientific) and the Direct-zol™ RNA Miniprep kit (Zymo Research, Irvine, CA). The resulting RNA concentrations were determined using a NanoDrop™ 2000c spectrophotometer (Thermo Fisher Scientific). SuperScript™ III Reverse Transcriptase (Thermo Fisher Scientific) was then used to generate complementary DNA (cDNA). Quantitative reverse transcription polymerase chain reaction (RT-PCR) assays were performed using SsoAdvanced™ Universal SYBR^®^ Green Supermix (Bio-Rad) and the CFX96 real-time polymerase chain reaction detection system (Bio-Rad), with the primers described in Supplementary Table S3. Relative expression levels were normalized to 18S ribosomal RNA (rRNA) expression, and fold changes in expression were calculated by the 2^*-ΔΔCT*^ method.

### NO assay

NO is rapidly oxidized to nitrite and nitrate, which are used to quantitate NO production. Nitrite and nitrate contents of serum and culture medium were measured by a spectrophotometric assay using the Nitric Oxide Assay Kit (Abcam, Cambridge, United Kingdom), according to the assay protocol. Briefly, a two-step process was performed, in which nitrate was first converted to nitrite utilizing nitrate reductase, followed by the use of Griess reagents to convert nitrite to a deep purple azo compound. Absorbance of the azo chromophore at 540 nm accurately reflects NO amounts in samples (Van Thuy et al, 2017).

### NO detection

Intracellular NO was evaluated with DAR-4M AM (GORYO Chemical, Inc., Hokkaido, Japan), a positively charged orange fluorescent probe that detects NO. Cells were incubated with DAR-4M AM (5 μ*M*) for 30 min at 37°C, and then washed with PBS (Furuuchi et al, 2018). The fluorescence signals were captured using a BZ-X700-All-in-One fluorescence microscope (Keyence Co., Osaka, Japan).

### Flow cytometry analysis of ROS and NO

Primary mouse HSC were seeded on 24-well plates (Greiner Bio-One International GmbH) in DMEM with 10% FBS. After 72 h, trypsinized cells were stained with CM-H_2_DCFDA (1:2000; Thermo Fisher Scientific) to evaluate intracellular ROS, or with diaminofluorescein-FM diacetate (1:2000; diaminorhodamine-FM diacetate [DAF-FM DA]; GORYO Chemical, Inc.), a positively charged green fluorescent probe to evaluate intracellular NO (Jozsef et al, 2002). Cells were analyzed using a BD™ LSR II flow cytometer (BD Biosciences, Franklin Lakes, NJ) (Urushima et al, 2021). NO detection was validated by multiple methods that support the highly specific data.

### Measurement of ROS in liver tissue

Liver tissue (200 mg) was homogenized in 2 mL of ice-cold 40 m*M* Tris-HCl buffer (pH 7.4) (Gabbia et al, 2018; Niknahad et al, 2017). To assess ROS levels, tissue homogenates (100 μL) were incubated for 40 min at 37°C with 1 mL of 10 μ*M* DCFDA solution (Sigma-Aldrich) diluted in Tris-HCl buffer. As a control for tissue autofluorescence, 100 μL of tissue homogenate was incubated with 1 mL of Tris-HCl buffer under the same conditions. Sample fluorescence intensities were assessed using the Varioskan™ LUX Multimode microplate reader at a wavelength range from 485 to 525 nm (Thermo Fisher Scientific).

### Isolation of mitochondria

After the gallbladder was removed using a scalpel, the liver was rinsed free of blood using ice-cold, degassed buffer for mitochondrial isolation (IBc), and was cut into small pieces using scissors (Frezza et al, 2007). The IBc used during the mincing was discarded and replaced with 5 mL of fresh ice-cold IBc, and the suspension was transferred to a 50 mL tube. Minced liver was homogenized using four strokes of a Teflon pestle at 1600 rpm, and the homogenate was transferred to a 50 mL tube and centrifuged at 600 *g* for 10 min at 4°C.

The supernatant was transferred to a new 50 mL tube and centrifuged at 7000 *g* for 10 min at 4°C. The supernatant was discarded, and the pellet was washed with 5 mL of ice-cold IBc and centrifuged at 7000 *g* for 10 min at 4°C. The supernatant was discarded, and the pellet containing mitochondria was resuspended in 50 m*M* Tris buffer (pH 7.4; Nippon Gene, Toyama, Japan). A portion of the pellet was used to measure mitochondrial concentration by protein assay.

### Isolation of hemoglobin

Mouse blood was collected with heparinized 1% NaCl solution. The red cells were washed several times with saline, and were hemolyzed by adding 10 volumes of water. The stroma was removed by centrifugation at 20,000 *g* for 30 min, and the Hb solution was dialyzed in the cold against 100 volumes of deionized water, frequently changed, for 48 h with gentle mechanical agitation. The concentration of Hb was measured using a NanoDrop One (Thermo Fisher Scientific).

### Activities of mitochondrial enzymes

Activities of mitochondrial enzymes were measured by a spectrophotometer at 37°C. In brief, complex I (NADH-ubiquinone oxidoreductase) activity was measured by monitoring the decrease in absorbance at 340 nm due to the oxidation of NADH to NAD, for 4 min. The reaction mixture contained 50 m*M* Tris buffer (pH 7.4), 1 m*M* potassium cyanide (KCN; Wako Pure Chemical Industries Ltd.), 100 n*M* antimycin A (Sigma-Aldrich), 60 μ*M* decylubiquinone (Sigma-Aldrich), and 100 μ*M* NADH (Sigma-Aldrich), with or without 200 n*M* rotenone. Complex II (succinate-ubiquinone oxidoreductase) activity was measured by monitoring the decrease in absorbance at 600 nm due to the reduction of 2,6-dichlorophenolindophenol (DCPIP), for 4 min. The reaction mixture contained 50 m*M* Tris buffer (pH 7.4), 1 m*M* KCN, 60 μ*M* DCPIP (Sigma-Aldrich), 60 μ*M* decylubiquinone, and 5 μ*M* succinate (Wako Pure Chemical Industries Ltd.), with or without 1 m*M* malonate (Wako Pure Chemical Industries Ltd.). Complex III (ubiquinol-cytochrome *c* oxidoreductase) activity was measured by monitoring the increase in absorbance at 550 nm due to reduction of cytochrome *c*, for 4 min. The reaction mixture contained 50 m*M* Tris buffer (pH 7.4), 1 m*M* KCN, 200 n*M* rotenone, 100 μ*M* decylubiquinol, and 75 μ*M* cytochrome *c* (Sigma-Aldrich), with or without 100 n*M* antimycin A. Complex IV (CcO) activity was measured by monitoring the decrease in absorbance at 550 nm due to the oxidation of reduced cytochrome *c*, for 4 min. The assay mixture consisted of 50 m*M* Tris buffer (pH 7.4) and 50 n*M* reduced cytochrome *c,* with or without 1 m*M* KCN. Lastly, citrate synthase activity was measured at 412 nm for 4 min in 50 m*M* Tris buffer (pH 7.4) containing 0.1 m*M* 5,5-dithiobis (2-nitrobenzoic acid) (Sigma-Aldrich), 0.3 m*M* acetyl-CoA lithium salt (Sigma-Aldrich), and 0.5 m*M* oxaloacetic acid (Sigma-Aldrich).

### Mitochondrial isolation and respirometry

Freshly excised livers were minced in 10 volumes of a mitochondrial isolation buffer (MHSE+BSA; 210 m*M* mannitol, 70 m*M* sucrose, 5 m*M* HEPES, 1 m*M* EGTA, and 0.5% [w/v] fatty acid-free BSA, with a pH 7.2), as described in Salabei et al (2014). The tissue was then homogenized using a Teflon glass homogenizer with six strokes. Mitochondria were isolated *via* differential centrifugation. The homogenate was centrifuged at 800 *g* for 10 min at 4°C, and the supernatant was decanted into a new tube. This fraction was centrifuged at 8000 *g* for 10 min to afford a mitochondrial pellet. The pellet was resuspended, and the centrifugation was repeated. The final pellet was resuspended in an MHSE+BSA, and a part of the pellet was used to measure mitochondrial concentration by the Bradford assay (TAKARA).

Mitochondria were pelleted in the cell culture miniplate *via* centrifugation at 2000 *g* for 20 min at 4°C, and the oxygen consumption rate was assessed using a Seahorse XF HS Mini Analyzer (Agilent Technologies). Mitochondrial respiration in a coupled state (10 μg/well) was measured in a mitochondrial assay solution (MAS; 220 m*M* mannitol, 70 m*M* sucrose, 10 m*M* KH_2_PO_4_, 5 m*M* MgCl_2_, 2 m*M* HEPES, 1 m*M* EGTA, and 0.2% [w/v] fatty acid-free BSA, with a pH 7.2 at 37°C) containing succinate as a substrate (10 m*M*) and rotenone (2 μ*M*). State 3 respiration (phosphorylating respiration) was triggered *via* the injection of 0.5 m*M* ADP. State 4 respiration was assessed by the addition of 3 *M* oligomycin, while maximal uncoupler-stimulated respiration was observed following the injection of 6 μ*M* carbonyl cyanide 4-(trifluoromethoxy) phenylhydrazone (FCCP).

At the end of the experiment, antimycin A (4 μ*M*), a complex III inhibitor, was added to inhibit mitochondrial respiration, as described in Tomas et al (2019). Liver mitochondria from WT and *Cygb*^−/−^ mice (*n* = 4) were included in each Seahorse analysis, and every sample was analyzed in triplicate.

### Detection of mitochondrial ROS

Mitochondrial ROS were measured at 37°C. The kinetic measurements were performed for 10 min with 10 μ*M* DCFDA solution and 100 μ*M* NADH. Fluorescence was recorded every 30 s using a Varioskan LUX Multimode Microplate reader at a wavelength range from 488 to 525 nm (Thermo Fisher Scientific).

### Measurement of CYP1A2 activity

CYP1A2 enzyme activity in primary mouse Hep was analyzed using the P450-Glo™ CYP1A2 assay system (Promega, Madison, WI) with Luciferin-ME as substrate, according to the manufacturer's protocol. The results were analyzed using a GloMax^®^ 96 Microplate Luminometer (Promega).

### Supercomplex purification

Mitochondria were solubilized in 100 m*M* sodium phosphate buffer (pH 7.4) containing 50 m*M* NaCl and 2% DDM (Dojindo Molecular Technologies, Inc., Tokyo, Japan) for 1 h at 4°C, and were centrifuged for 1 h at 100,000 *g* at 4°C. The supernatant was loaded onto 0.5–1.5 *M* sucrose gradients in buffer containing 100 m*M* HEPES, pH 7.4, 20 m*M* KCl, and 0.01% DDM, and centrifuged at 150,000 *g* for 21 h at 4°C. Gradients were fractionated and evaluated by clear-native PAGE.

### Clear-native PAGE

Anode buffer for clear-native PAGE contained 25 m*M* imidazole HCl, pH 7.0, and cathode buffer contained 50 m*M* Tricine, 7.5 m*M* imidazole, 0.02% (w/v) DDM, and 0.05% (w/v) sodium deoxycholate (Wittig et al, 2007). Electrophoretic separations on 4%–15% polyacrylamide Mini-PROTEAN^®^TGX™ Precast Gels (Bio-Rad) were carried out at room temperature, at a constant voltage of 150 V for 2 h, using a KS-8001 electrophoresis chamber (Oriental Instruments. Co., Ltd., Kanagawa, Japan). After electrophoresis, the proteins were visualized by Coomassie staining.

### Measurement of AST and ALT

AST and ALT in serum were measured using a commercially available kit (Wako Pure Chemical Industries Ltd.), according to the manufacturer's protocol.

### Statistics and reproducibility

All experiments were replicated at least three times. ImageJ was used to evaluate Western blot band intensities (National Institutes of Health). Differences among experimental groups were analyzed using unpaired Student's *t*-tests or one-way analysis of variance (ANOVA) or two-way ANOVA, performed using GraphPad Prism 6 software (La Jolla, CA). Values of *p* < 0.05 were considered statistically significant. The data are displayed as the mean ± standard deviation.

## Supplementary Material

Supplemental data

Supplemental data

Supplemental data

Supplemental data

## Abbreviations Used

αSMA: α-smooth muscle actin
ALT: alanine aminotransferase
AMED: Japan Agency for Medical Research and Development
ANOVA: analysis of variance
AST: aspartate aminotransferase
CcO: cytochrome *c* oxidase
CM: conditioned medium
CYGB: cytoglobin
*Cygb* ^-/-^: *Cygb* knockout
CYP1A2: cytochrome P450 1A2
Cyt *c*: cytochrome *c*
DAR-4M AM: diaminorhodamine-4M acetoxymethyl ester
DCPIP: 2,6-dichlorophenolindophenol
DDM: n-dodecyl-β-D-maltopyranoside
DMEM: Dulbecco's modified Eagle's medium
e^-^: electron
ECM: extracellular matrix
eNOS: endothelial nitric oxide synthase
ETC: electron transport chain
FBS: fetal bovine serum
FCCP: carbonyl cyanide 4-(trifluoromethoxy) phenylhydrazone
GAPDH: glyceraldehyde-3-phosphate dehydrogenase
Hb: hemoglobin
HbO_2_: oxyhemoglobin
HCC: hepatocellular carcinoma
Hep: hepatocytes
HSC: hepatic stellate cells
iNOS: inducible nitric oxide synthase
KCN: potassium cyanide
L-NAME: N^ω^-nitro-L-arginine methyl ester hydrochloride
LSEC: liver sinusoidal endothelial cell
MFI: mean fluorescence intensity
Mito: mitochondria
mRNA: messenger RNA
mtDNA: mitochondrial DNA
NADH: nicotinamide adenine dinucleotide
ND: not detected
NO: nitric oxide
NOD: nitric oxide dioxygenase
NOS: nitric oxide synthase
ns: not significant
O_2_: oxygen
PAGE: polyacrylamide gel electrophoresis
PBS: phosphate-buffered saline
Q: ubiquinone
QH_2_: ubiquinol
RCR: respiratory control ratio
RFU: relative fluorescence units
RLU: relative light units
ROS: reactive oxygen species
SD: standard deviation
TGF-β: transforming growth factor-beta
WT: wild type
