# Supplementary material for: Nitric Oxide Derived from Cytoglobin-Deficient Hepatic Stellate Cells Causes Suppression of Cytochrome c Oxidase Activity in Hepatocytes
Source: Antioxid Redox Signal. 2023 Mar 16;38(7-9):463–79. doi: 10.1089/ars.2021.0279 (PMC10025843; doi:10.1089/ars.2021.0279)
Supplement: Supplemental data [file Suppl_TableS1.pdf]

Supplemental Table 1. Purification of cytochrome *c* oxidase

WT mice	Total protein (mg)	Specific activity (μmol/min/mg)	Recovery (%)	k_{cat} (s^{-1})
Mito^{WT}	51.5	0.24	100	
Solubilization	61.9	0.18	92.3	
Purified fraction	1.02	5.32	44.3	17.5

Cygb$^{-/-}$ mice	Total protein (mg)	Specific activity (μmol/min/mg)	Recovery (%)	k_{cat} (s^{-1})
Mito$^{Cygb-/-}$	52.5	0.17	100	
Solubilization	69.1	0.13	99.4	
Purified fraction	1.17	4.28	56.7	14.3
