# Supplementary figures and images for: Nitric Oxide Derived from Cytoglobin-Deficient Hepatic Stellate Cells Causes Suppression of Cytochrome c Oxidase Activity in Hepatocytes
Source: Antioxid Redox Signal. 2023 Mar 16;38(7-9):463–79. doi: 10.1089/ars.2021.0279 (PMC10025843; doi:10.1089/ars.2021.0279)

# Supplementary Figure

## uncropped raw image

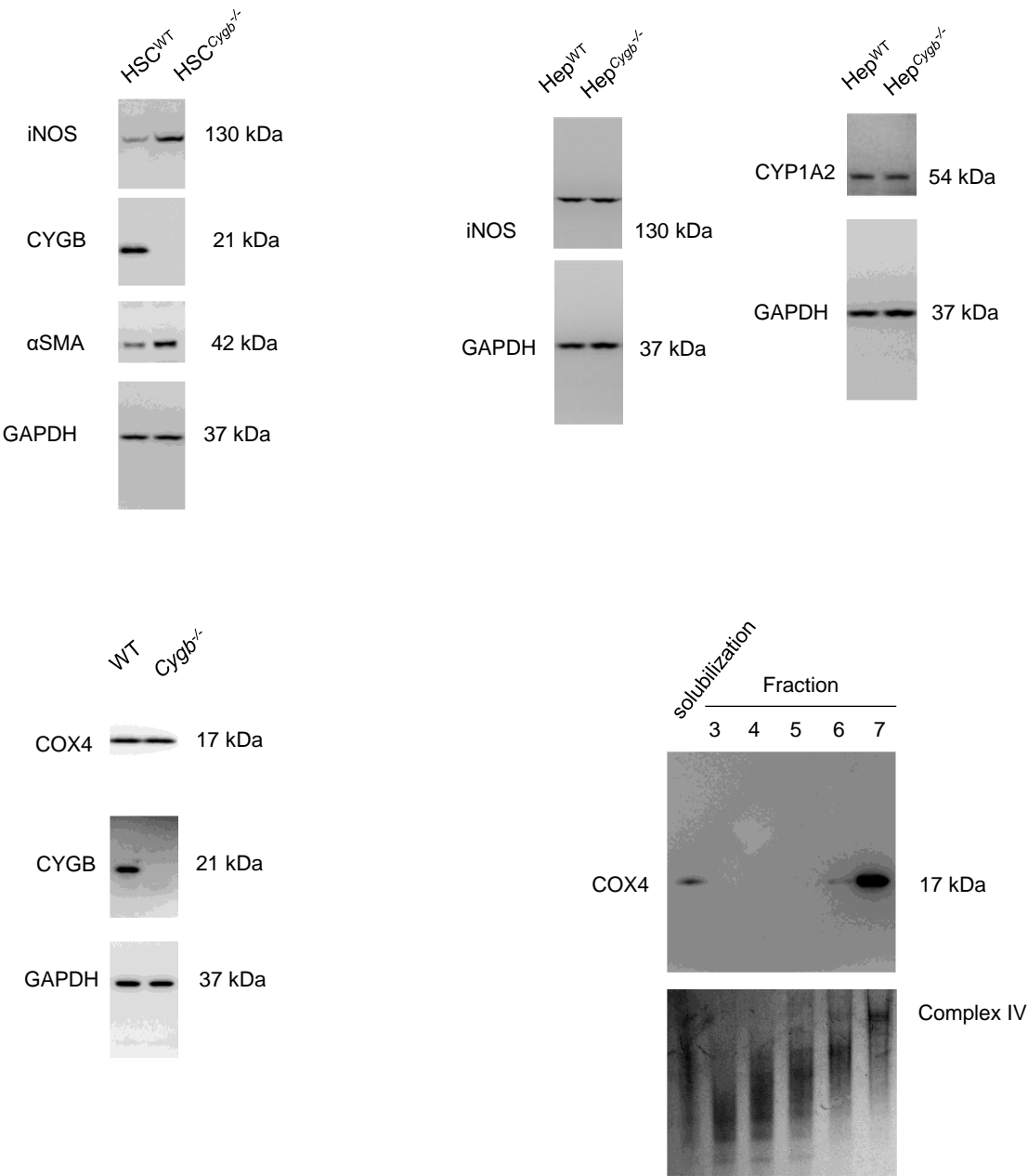

Supplement: Supplemental data [file Suppl_FigureS1.pdf]
