# Supplementary material for: Nitric Oxide Derived from Cytoglobin-Deficient Hepatic Stellate Cells Causes Suppression of Cytochrome c Oxidase Activity in Hepatocytes
Source: Antioxid Redox Signal. 2023 Mar 16;38(7-9):463–79. doi: 10.1089/ars.2021.0279 (PMC10025843; doi:10.1089/ars.2021.0279)
Supplement: Supplemental data [file Suppl_TableS2.pdf]

Supplemental Table 2. Antibodies used in the study

anti-CYP1A2 (D15)	mouse	monoclonal	Santa Cruz Biotechnology (sc-53241)	1/1000
anti-mouse Cygb	rabbit	polyclonal	Our laboratory	1/1000
anti-GAPDH (clone 6C5)	mouse	monoclonal	Millipore	1/10000
anti-NOS2 (C-11)	mouse	monoclonal	Santa Cruz Biotechnology (sc-7271)	1/1000
anti-COX4	mouse	monoclonal	Invitrogen (A21348)	1/1000
