# Supplementary material for: Nitric Oxide Derived from Cytoglobin-Deficient Hepatic Stellate Cells Causes Suppression of Cytochrome c Oxidase Activity in Hepatocytes
Source: Antioxid Redox Signal. 2023 Mar 16;38(7-9):463–79. doi: 10.1089/ars.2021.0279 (PMC10025843; doi:10.1089/ars.2021.0279)
Supplement: Supplemental data [file Suppl_TableS3.pdf]

Supplemental Table 3. Primers used for quantitative RT-PCR

Gene name	Forward	Reverse
mCygb	CCTCCATGTGTCTAAACTGGC	GCTGTATGCCAACTGCGAG
mαSma	GTTCAGTGGTGCCTCTGTCA	ACTGGGACGACATGGAAAAG
mCol1a1	TAGGCCATTGTGTATGCAGC	ACATGTTCAGCTTTGTGGACC
mNos2	TGAAGAAAACCCCTTGTGCT	TTCTGTGCTGTCCCAGTGAG
mNos3	CCTAGAGCACGAGGCACTG	GTTGTACGGGCCTGACATTT
mCox2	AACCGAGTCGTTCTGCCAAT	CTAGGGAGGGGACTGCTCAT
mCox4	GCCTTGGACGGCGGAAT	CCACATCAGGCAAGGGGTAG
mCyp1a2	ACAGCAAGGACTTTGTGGAGAA	ACAGGGCACTTGTGATGTCTT
m18s	CGGCTACCACATCCAAGGAA	ATTGGAGCTGGAATTACCGC

‘m’ indicates mouse cDNA-specific primer sequences.
